# Intensity Interrogation-Based High-Sensitivity Surface Plasmon Resonance Imaging Biosensor for Apoptosis Detection in Cancer

**DOI:** 10.3390/bios13100946

**Published:** 2023-10-23

**Authors:** Xin Yuan, Zhenxiao Niu, Lang Liu, Youjun Zeng, Lin Ma, Zhaogang Nie, Zhen Tian, Dongyun Kai, Fangteng Zhang, Guanyu Liu, Siwei Li, Zhengqiang Yuan

**Affiliations:** 1 School of Biomedical and Pharmaceutical Sciences, Guangdong University of Technology, Guangzhou 510006, China; 2112112060@mail2.gdut.edu.cn (X.Y.); 2112112055@mail2.gdut.edu.cn (L.L.); 2 School of Physics & Optoelectronic Engineering, Guangdong University of Technology, Guangzhou 510006, China; 17793175617@163.com (Z.N.); malin@gdut.edu.cn (L.M.); zgnie@gdut.edu.cn (Z.N.); 2112115091@mail2.gdut.edu.cn (D.K.); zhang.ft@gdut.edu.cn (F.Z.); liuguanyulh@163.com (G.L.); 3 School of Physical Science and Information Technology, Liaocheng University, Liaocheng 252059, China; zhentian166@163.com; 4 School of Mechano-Electronic Engineering, Zhuhai City Polytechnic, Zhuhai 519000, China; zhcptlsw@163.com

**Keywords:** SPRi, caspase-3, apoptosis, cancer, therapeutic efficacy assessment

## Abstract

Intensity interrogation-based surface plasmon resonance imaging (ISPRi) sensing has a simple schematic design and is the most widely used surface plasmon resonance technology at present. In this study, we report the successful development of a novel high-sensitivity ISPRi biosensor and its application for apoptosis detection in cancer cells. By optimizing the excitation wavelength and excitation angle, we achieved a refractive index resolution (RIR) of 5.20 × 10^−6^ RIU. Importantly, the biosensor has been tested and validated for high-throughput and label-free detection of activated caspase-3 with its specific inhibitor Z-DEVD-FMK in apoptotic cells. Therefore, this study describes a novel molecular imaging system to monitor apoptosis in cancers for disease diagnosis and/or evaluation of therapeutic efficacy of anti-cancer drugs.

## 1. Introduction

Apoptosis is a programmed cell death triggered by intrinsic and/or extrinsic stimuli. The molecular imaging and tracking of apoptosis can be utilized for drug screening or assessment of therapeutic efficacy of anticancer drugs. As one of the most prominent executioners of apoptosis, the cleaved (activated) caspase-3 (C-Casp-3) plays a crucial role in the apoptotic signaling pathway. Routine C-Casp-3 detection techniques, like Western blotting and immunostaining, come with challenges such as selectivity, antibody cost, and transient caspase-3 activation.

Comparing to the aforementioned biological detection methods, surface plasmon resonance (SPR), which can perform label-free, high-sensitivity, and real-time detection, has become an attractive approach for analyzing biomolecular interactions [1,2,3,4]. SPR has been applied in a number of fields, such as biological detection and inspection of food safety [5,6,7,8]. In addition, by combining imaging technology, the SPR imaging (SPRi) sensing technology can achieve parallel monitoring of multiple samples [1,9]. To date, four interrogation SPRi modes have been proposed, including intensity-, angle-, wavelength-, and phase-interrogation modes [10]. Of these, the intensity interrogation SPRi (ISPRi) has the advantage of structural simplicity and easy implementation, and it is currently the most commonly used modulation mode for commercial SPRi sensing instruments [11].

The ISPRi biosensor usually fixes the incidence wavelength and angle, and it can monitor sample change by detecting the reflected light intensity. Nevertheless, the refractive index resolution (RIR) of ISPRi sensors is usually limited to the 10^−5^ RIU order [10]. A narrowband monochromatic laser can be used as an incident light source to excite the SPR phenomenon, thus achieving improved sensing. Laser has the advantages of good monochromaticity and high brightness, which can improve the signal-to-noise ratio (SNR) of the system. However, the laser speckle causes speckling noise and reduces the imaging quality [12,13]. Therefore, the laser source has certain intrinsic limitations for imaging sensing.

In order to improve sensing performance, Zybin et al. proposed a double-wavelength SPR interrogation strategy to achieve both high-throughput monitoring capacity and high sensitivity [14]. They utilized two diode-lasers to obtain differential measurements, while employing a photo-diode matrix as the detector. Their system allows for simultaneous monitoring of four channels and achieves a refractive index resolution (RIR) of 5 × 10^−6^ RIU. However, speckles induced by two coherent light sources can introduce significant noises, particularly when it comes to intensity-based SPR sensing. Similarly, we previously constructed a highly sensitive ISPRi sensor [15]. Moreover, the system employs a halogen lamp as the excitation light source to prevent speckle noise and to improve imaging quality. Our approach focuses on interrogating the differential values of two intensities at two specific wavelengths derived from the reflected light spectrum. Furthermore, we employed a halogen lamp as the excitation light source in our system to effectively mitigate speckle noise and enhance the overall imaging quality. With these optimizations, our sensor achieved an impressive RIR of 2.24 × 10^−6^ RIU. However, the above-mentioned dual receiving optical path structure is complex and requires correction for different sensing sites. Additionally, for prism coupling structure, it is difficult to adjust the optical angle during the imaging sensing process due to the varying degrees of distortion caused by different incident angles.

In this study, we developed a simplified ISPRi biosensor with high sensitivity by optimizing the excitation wavelength and incident angle. This biosensor was tested and validated for reliable performance via the detection and analysis of the activation level of caspase-3 in cancer cells with label-free and high-throughput features, which can be used to monitor and assess apoptosis induction by anti-cancer drugs treatment at the molecular level. 

## 2. Establishment of Biosensing System

### 2.1. Both the Excitation Wavelength and Incidence Angle Were Optimized

In this section, we will analyze the impact of the incident wavelength and angle on the system, considering that the system maintains a fixed incident wavelength and angle [16]. By utilizing incoherent light sources, such as tritium lamps, halogen lamps, or LEDs, the speckle effect generated by lasers can be avoided, resulting in improved imaging quality and signal-to-noise ratio (SNR) in the sensing system. These incoherent light sources commonly operate within the visible to near-infrared wavelength ranges.

**Figure 1:**
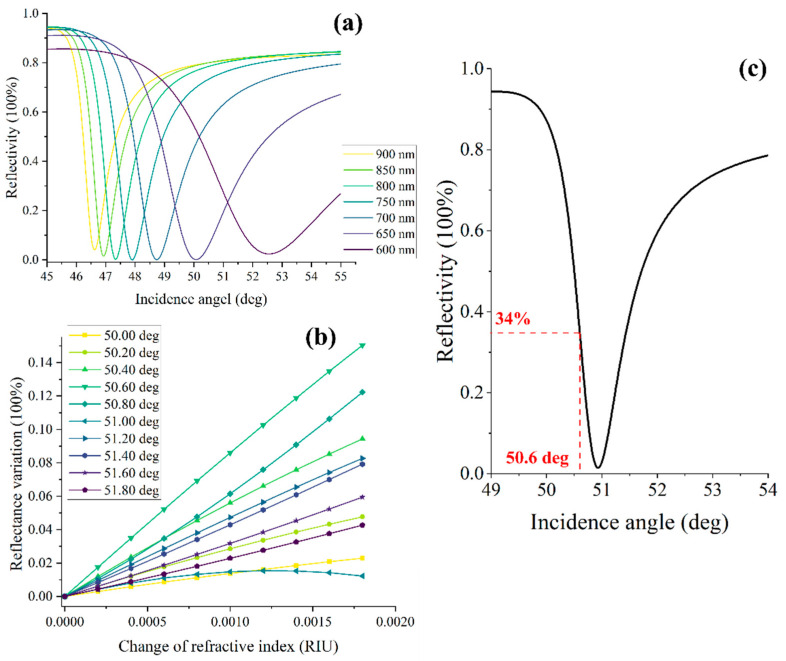
Theoretical calculation and optimization of incident angle and wavelength. (**a**) The observed SPR angle spectrum curves with different incidence wavelengths; (**b**) the reflectivity varies with samples at different incident angles at 850 nm incident wavelength; (**c**) the obtained SPR angular spectrum curve under 850 nm incident wavelength with the sample refractive index of 1.333 RIU.

In order to optimize the incident wavelength, we simulated the SPR angular spectrum curves at various incident wavelengths (Figure 1a). The simulation parameters are the following: a 48 nm-thick layer of gold as the sensing chip, a refractive index of 1.785 RIU for the coupling prism, and a sample refractive index of 1.333 RIU. Notably, the obtained results showed that the near-infrared band exhibited a narrower full width at half maximum (FWHM) compared with the visible light. This finding suggests that the biosensor system can achieve higher sensitivity when employing the near-infrared band as the excitation light source [17,18]. Additionally, the incident angle plays a crucial role in the performance of the ISPRi system. We analyzed the reflectivity with different incident angles for an incident wavelength of 850 nm. The refractive index of the sample varied incrementally from 1.333 to 1.335 with a step size of 0.0002 RIU (Figure 1b). Remarkably, the obtained signal exhibited a maximum response curve with good linearity when the incident angle of 51.6 deg was employed. Additionally, when the refractive index of the sample was set to 1.333, the angular spectrum curve showed a reflectivity of 34% at the corresponding incident angle of 5.16 deg (Figure 1c). During the actual testing process, the optimal incident angle can be determined by adjustment to achieve a corresponding reflectivity of 34%.

### 2.2. System Setup

**Figure 2:**
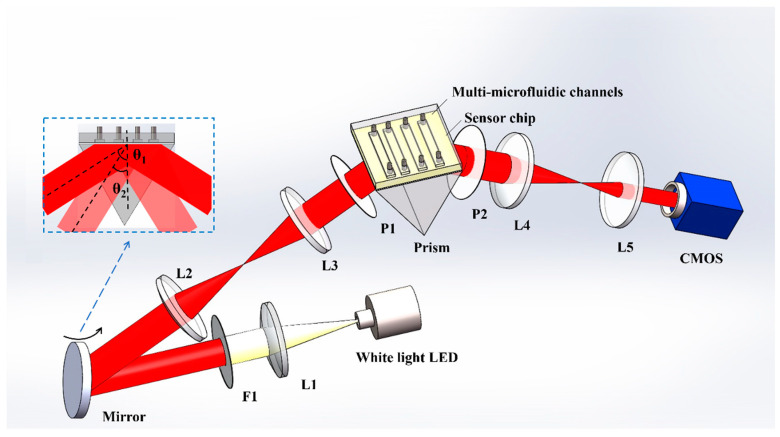
Optical setup. L1–5: lens; P1–2: polarizer; F1: filter.

The ISPRi biosensor is shown in Figure 2. The system utilizes an LED (GCI-060401, Daheng Optics, Beijing, China) as the excitation light source. The white light passes through various components before reaching the sensing module. First, it passes through the collimating lens L1 (with a focal length of 150 mm), followed by filter F1 (with a center wavelength of 850 nm and a FWHM of 10 nm). This configuration ensures that only parallel narrowband light is incident on the rotating mirror, which allows the selection of the incident angle. Lens groups L2 (with a focal length of 60 mm) and L3 (with a focal length of 60 mm) form a 4f system. The reflector is positioned on the front focal plane, while the sensing surface is located on the rear focal plane. The sensing module comprises a prism, a sensing chip, and a flow cell. Then, the incidence light passes the polarizer P1 (p-polarized), and it is coupled through the prism to excite the surface plasmon resonance (SPR) phenomenon at the interface between the sensing chip and the detection sample. The reflected light then passes through imaging lens groups L4 (with a focal length of 200 mm), L5 (with a focal length of 75 mm), and polarizer P2 (p-polarized). Finally, it is received by the area array detector CMOS (DMK 33GP031, Imaging Source, Bremen, Germany). 

Lens groups L4 and L5 form another 4f system, with the sensing surface and the CMOS film positioned on the front and rear focal surfaces of the system, respectively. As shown in the small blue dashed box in Figure 2, in the incident light path, the incidence angle can be adjusted by rotating the reflector. Different angles (e.g., θ_1_ and θ_2_) of incident light pass through the 4f system (composed of L2, L3) and prism, and then enter the sensing surface. Due to the distortion caused by the prism, the size of the incident light spot on the sensing surface changes. However, due to the presence of the 4f system, its central position does not change. In the reflected light path, the 4f system can ensure that the light spot on the sensing surface is always received by the CMOS, and the relative position between the sensor surface and photographic film of CMOS does not change. The utilization of these two 4f systems ensures that the center position of the imaging detection area remains unchanged during the angle adjustment process. The reflected light intensity at each point on the sensing surface is continuously monitored in real time using the CMOS, which is controlled by a customized Labview program.

## 3. Results and Discussion

### 3.1. Dynamic and RIR

**Figure 3:**
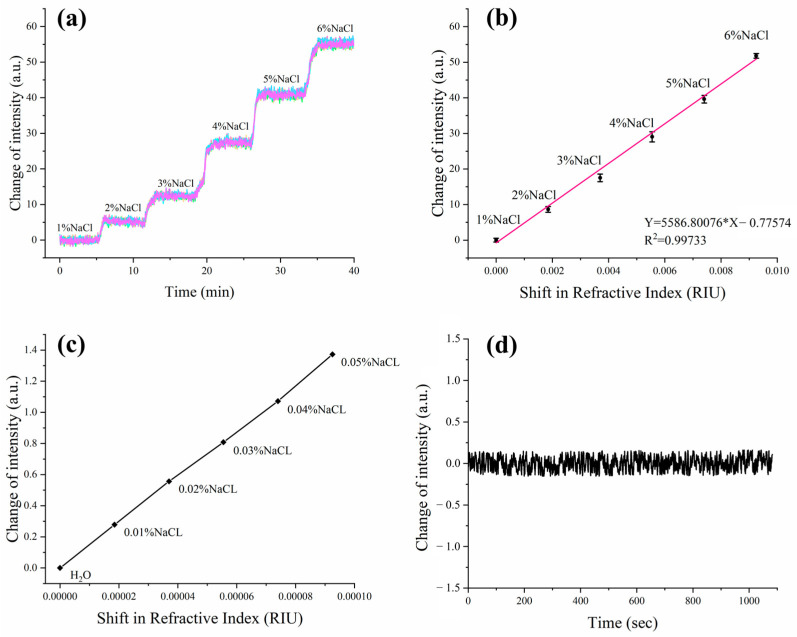
Examination of ISPRi system performance parameters using various concentrations of NaCl. (**a**) The detected real-time shift of SPR signal over NaCl concentration changes among the 3 × 3 array of detection sites on the sensor chip; (**b**) the curve of SPR signal intensity over refractive indexes on the 3 × 3 array of sensors; (**c**) SPR signal shift obtained from low concentrations NaCl from 0% to 0.05%; (**d**) examination of the SPR signal shift with pure water for a period of 10 min.

The ISPRi sensor’s capabilities were first demonstrated through meticulous experimentation, as we measured the intensity shift among different concentrations of NaCl solution. The NaCl solutions were carefully prepared, encompassing concentration levels ranging from 0% to 5% in precise 1% increments by volume. These concentrations correspond to refractive indices spanning from 1.3330 to 1.3423 RIU. During the experiment, the incidence angles were carefully adjusted to achieve a reflectivity of 34% by using pure water, and subsequently the angle was maintained in a fixed position. Different concentrations of NaCl solution were injected into four distinct channels to examine the possible SPR signal shift. The obtained dynamic response curves are shown in Figure 3a, which demonstrates the system’s good performance for multi-channel real-time parallel detection. 

The SPR signal shift was also visually demonstrated by the changes in the refractive index in samples (Figure 3b). It is noteworthy that these observations revealed a robust linear relationship between the SPR signal intensity and the refractive index. The dynamic range of the established detection system was determined as 0.00000–0.00925 RIU, following the previously reported calculation method [19,20]. Additionally, the results also show that the system has good consistency for multi-points sensing. Low concentrations of saline were tested for corresponding RIR (Figure 3c). Increasing saline concentrations were tested from 0% to 0.05% with 0.01% for each increment. These examinations enable us to precisely determine the system’s resolution and its ability to accurately detect even minute changes in the refractive index. Furthermore, to assess the system’s noise characteristics, we conducted tests for continuous and real-time detection of reflected light signal intensity in pure water for a duration of 10 min. As shown in Figure 3d, the obtained results provide valuable insights into the system’s stability and reliability.

(1) $$\sigma_{RI}=\frac{\delta n}{\delta s}\Delta \sigma_{SD},$$

According to the above Formula (1) [6,21], the RIR of the system can be calculated as 5.20 × 10^−6^ RIU, where $\sigma_{RI}$ is the sensitivity, δn is the refractive index change, δs is the change in intensity, and $\Delta \sigma_{SD}$ is the root mean square (RMS) noise of the ISPRi system. In this case, the values of δn, δs, and $\Delta \sigma_{SD}$ are given as 0.0000925 RIU, 1.37 a.u., and 0.077 a.u., respectively.

### 3.2. SPRi Detection of Apoptosis in Cancer Cells with a Caspase-3 Inhibitor 

#### 3.2.1. Materials and Reagents

##### Chemicals and Materials

Phosphate-buffered saline (PBS, pH 7.4) and bovine serum albumin (BSA) were purchased from Gibco (Life Technologies, Gaithersburg, MD, USA). Refractive index matching oil with a refractive index 1.780 RIU was obtained from Cargille Laboratories, Inc. (Cedar Grove, NJ, USA). NaCl was purchased from Aladdin (C111535-500g, GR99.8%, Shanghai, China). The gold film in the sensing chip is based on 1 mm-thick glass (length: 18 mm, width: 18 mm; SF11 material with a refractive index of 1.785) as the substrate, with a 48 nm-thick gold layer being deposited on the substrate through evaporation, and 2 nm of chromium layer spacing between the glass substrate and the gold coating to increase the fixation effect.

##### Cell Culture and Reagents

Cell culture plates/flasks were purchased from Thermo Fisher Scientific (Guangzhou, China), and reagents were obtained from Gibco (Grand Island, NY, USA). The human lung cancer line A549 cell line was obtained from Shanghai FUHENG Biological Technology Co., Ltd., and cultured in RPMI 1640 medium containing 10% fetal bovine serum (FBS) at 37 °C in a humidified incubator with supplement of 5% CO_2_. The highly specific inhibitor of cleaved/activated caspase-3 (C-Casp-3), Z-DEVD-FMK (HY12466, Shanghai, China) was purchased from MedChemExpress (MCE) and dissolved in DMSO at 20 mM as the stock solution. Tumor necrosis factor (TNF)-related apoptosis-inducing ligand (TRAIL)-expressing extracellular vesicles (EV-Ts) that carry membranous TRAIL for efficient killing of cancer cells were obtained from a previous study [22]. Dinaciclib (Dina), a potent cyclin-dependent kinase (CDK) inhibitor (SCH727965) [23], was obtained from Selleck Chemicals (Shanghai, China), and dissolved in DMSO for the preparation of 10 mM stock solution. EV-Ts and Dina were shown to be synergistic for inducing apoptosis in A549 cells previously [22], and these two drugs were used for combination treatment of A549 cells in this study. 

##### Apoptosis Assay by Annexin V/PI Staining and Flow Cytometry

For apoptosis assay, cells were seeded in 6-well plates at a density of 4 × 10^5^ cells/well. After settling down for 12 h, cells were treated by vehicle PBS or therapeutic drugs for 24 h. Subsequently, both adherent and floating cells were harvested, washed, and labeled with FITC-conjugated Annexin V and propidium iodide (Bestbio, Shanghai, China) for detection of apoptotic cells according to the manufacturer’s instructions. Next, the labeled cells were analyzed to determine their apoptosis rate via flow cytometry (FACS Caliber, Becton Dickinson, Franklin Lakes, NJ, USA). Annexin V+/PI− cells are regarded as in an early apoptosis stage, whilst annexin V+/PI+ cells are in a late apoptosis state, and annexin V−/PI− cells are alive and annexin V−/PI+ labeling suggests non-apoptotic cell death.

##### Western Blotting

Western blotting was performed to detect both caspase-3 (CASP-3) zymogen and cleaved/activated caspase-3 (C-CASP-3) in cellular lysates according to a previous description [24]. In brief, A549 cells were first lysed in radio-immunoprecipitation assay (RIPA) buffer containing complete protease inhibitor cocktail (Complete-mini; Roche Diagnostics GmbH, Mannheim, Germany) for extraction of total cellular proteins. Then, the total protein concentrations in cellular lysates were measured by using the BCA assay (Beyotime Biotech, Guangzhou, China). Fifteen µg of total proteins for each sample were run in SDS-PAGE for separation, electro-transferred to polyvinylidene fluoride (PVDF) membranes (Roche Diagnostics), and then membrane blocked for 1 h in TBST buffer containing 10% skimmed milk. Subsequently, the membrane was incubated overnight at 4 °C with primary antibodies (Abs) against the following proteins: CASP-3 (Abcam, Cambridge, UK, ab32351, 1:1000 dilution), C-CASP-3 (Proteintech, Hong Kong, China, 25128-1-AP, 1:1000 dilution), and GAPDH (Affinity, West Bridgford, UK, AF7021, 1:3000 dilution). After primary Abs incubation, the membranes were washed and incubated with horseradish peroxidase (HRP)-conjugated secondary Abs (1:10,000), developed for chemiluminescence using an ECL detection kit (Merck Millipore, Burlington, MA, USA) and imaged with a BioImage Lab device (Bio-Rad, Hercules, CA, USA). Western blotting bands were quantitated by using the Image J software (Version number: 1.52v, National Institutes of Health, Bethesda, MA, USA) with GAPDH as an internal loading control. The expression levels of target proteins are shown relative to the control sample, for which the value was set to 1.

#### 3.2.2. ISPRi Detection of Cleaved Caspase-3 (C-CASP-3) with Its Inhibitor Z-DEVD-FMK

Harvested cells were lysed in RIPA buffer supplemented with protease inhibitor cocktail for extraction of total cellular proteins. 

For the pretreatment of the sensing chip, the total cellular proteins were bound onto the gold film of the chip as bio probes. The sample channels were first rinsed with PBS. Once SPR signal was stabilized, 140 µg of total cellular proteins from control or drug treatment groups were diluted with PBS and injected into the flow cells, allowing the proteins to fix on the sensing chip through physical adsorption [15]. For blank control, only PBS was injected. Subsequently, the chip was flushed via PBS washing, and blocked with 100 µg/mL of BSA for 5 min at room temperature (RT) to prevent nonspecific binding of inhibitor to the chip surface. Then, PBS was injected to each channel for 5 min to obtain the baseline for the following detection of molecular interaction between the cleaved caspase-3 (C-CASP-3) and its inhibitor Z-DEVD-FMK. The inhibitor Z-DEVD-FMK at 20 nM was loaded to the sensor chip for binding to C-CASP-3 that was contained in the cell lysate proteins for 5 min at RT. Thereafter, PBS was injected into the channels to wash away the nonspecifically bound inhibitor. Then, the refractive light intensities could reach a stable value again, reflecting possible molecular interactions on the chip surface.

#### 3.2.3. Detection of Activated Caspase-3 with Its Inhibitor Z-DEVD-FMK by ISPRi Biosensor

The performance of the ISPRi sensor was further evaluated by detecting the expression levels of cleaved/activated caspase-3 (C-Casp-3) with its specific inhibitor Z-DEVD-FMK in A549 lung cancer cells. Caspases (Cysteine-aspartic proteases) function as proteases and are involved in cellular apoptosis [25]. All caspases are biosynthesized as inactive forms and subdivided into two types of zymogens, i.e., initiator caspases, such as caspase-8 and -9, and executioner caspases, such as caspase-3 and -6. Once activated by apoptotic signaling, the initiator caspases function to cleave and activate executioner caspases, and the activated executioner caspases cleave hundreds of proteins substrates, leading to apoptosis in cells [26,27]. Caspase-3 is an essential executioner of apoptosis, and its active expression level can be thus employed to reveal apoptosis status in cells [24,28]. As an irreversible caspase-3 inhibitor, the peptide Z-DEVD-FMK specifically binds to C-Casp-3 and suppresses its proteolytic activity [29]. 

The chemo drug Dina was previously found to synergize with TRAIL-expressing EVs (EV-Ts) to efficiently induce apoptosis in A549 cells [22,30]. In this study, we first treated A549 cells for apoptosis induction by combining the previously prepared EV-T with Dina (EV-T-Dina). Then, we harvested both control and drug-treated cells to extract total cellular proteins for detection of C-CASP-3 with its specific inhibitor Z-DEVD-FMK by using the constructed ISPRi system. 

**Figure 4:**
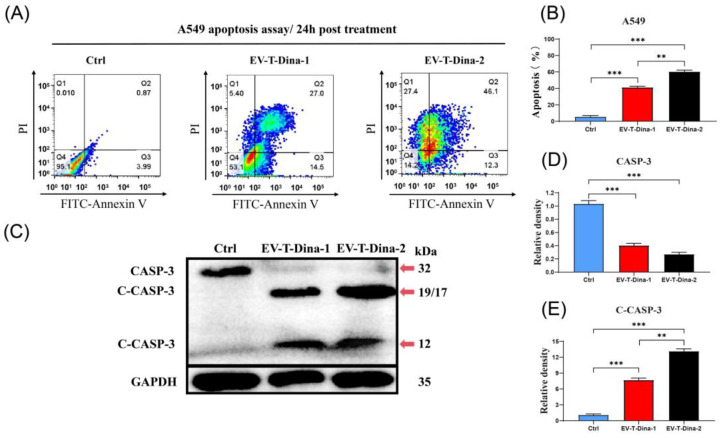
Treatment with EV-T-Dina stimulated significant apoptosis in A549 cells. (**A**) Representative flow cytometry analysis plots for assessing apoptosis in A549 cells after FITC-Annexin V/PI staining. Cells were treated with PBS (Ctrl), 1.0 ng mL^−1^ EV-T TRAIL + 10.0 nM Dina (EV-T-Dina-1), and 2.0 ng mL^−1^ EV-T TRAIL + 20.0 nM Dina (EV-T-Dina-2) for 24 h before the evaluation, respectively. (**B**) Comparison of apoptosis rates among control and treatment groups. (**C**) Detection of both caspase-3 (CASP-3, 32 kDa) and cleaved/activated caspase-3 (C-CASP-3, 19/17- and 12- kDa) expression in A549 cells via Western blotting. (**D**,**E**) Relative quantification of both CASP-3 (**D**) and C-CASP-3 ((**E**), 19/17 kDa) expression levels among control and EV-T-Dina treatment groups. All data are means ± SD (*n* = 3). ** *p* < 0.01, *** *p* < 0.001, by one-way ANOVA/Bonferroni multiple comparison post hoc test.

As shown in Figure 4A, EV-T-Dina treatment induced significant apoptosis in A549 cells. Notably, the higher concentration of drug (EV-T-Dina-2) treatment led to a significantly higher apoptosis induction rate than the lower dose (EV-T-Dina-1) (Figure 4B). Both CASP-3 and C-CASP-3 were detected through immunoblotting in A549 cell lysates that contain soluble cellular proteins. The obtained results showed that EV-T-Dina treatment resulted in the cleavage/activation of CASP-3, thus reducing the expression of CASP-3 (32 kDa band), but increasing the expression of C-CASP-3 (19/17- and 12-kDa bands) (Figure 4C,D). Furthermore, the higher drug dose induced significantly higher C-CASP-3 expression levels (Figure 4E). 

Four channels were set on the sensor chip, including blank control (Blank), no drug-treatment control (Ctrl), low and high dose of EV-T-Dina treatments (EV-T-Dina-1 and EV-T-Dina-2). The Blank control channel was not primed with cellular proteins, but Ctrl and EV-T-Dina channels were primed with control cell proteins and drug treated cell proteins, respectively. After PBS washing, all channels were introduced with the C-CASP-3 inhibitor to examine C-CASP-3/inhibitor interaction-related SPR signal changes. 

**Figure 5:**
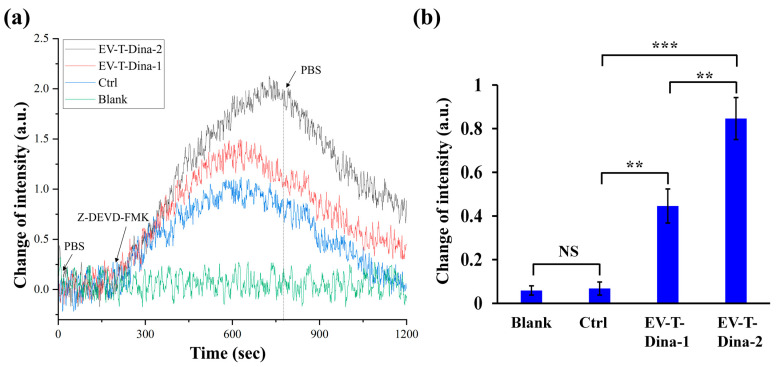
The real-time SPRi detection of biomolecular interaction between C-CASP-3 and its inhibitor Z-DEVD-FMK. (**a**) Representative SPRi curves for four-channel detection. The four SPRi channels were injected with PBS (Blank), total cellular proteins from control A549 cells without drug treatment (Ctrl), or total cellular proteins extracted from A549 cells treated with 1 ng/mL EV-T TRAIL and 10 nM Dina (EV-T-Dina-1) or treated by 1 ng/mL EV-T TRAIL and 20 nM Dina (EV-T-Dina-2), respectively. (**b**) Comparison of stable SPRi signal intensity changes before and after inhibitor loading among the four detection channels. All values are means ± SD (*n* = 3). NS, not significant, ** *p* < 0.01, *** *p* < 0.001, via one-way ANOVA/Bonferroni multiple comparison post hoc test.

As shown in Figure 5a, three of the channels showed some extent of signal increases after the inhibitor introduction, due to the difference in the refractive index between the PBS and inhibitor solution and/or the molecular interaction between C-CASP-3 and its inhibitor. However, after PBS washing and signal stabilization, both Blank and Ctrl channels returned to the baseline signal levels, whilst both EV-T-Dina channels demonstrated significant signal intensity increases when compared with the Ctrl (Figure 5a,b). Additionally, the EV-T-Dina-2 channel showed significantly higher signal change than the EV-T-Dina-1 one, reflecting the fact that the EV-T-Dina-2 cells expressed a higher level of C-CASP-3 than the EV-T-Dina-1 cells. Collectively, these data suggest that the established ISPRi system is competent and reliable for detecting activated caspase-3 expression, and thus can be employed to evaluate anti-cancer drug therapeutic efficacy.

**Figure 6:**
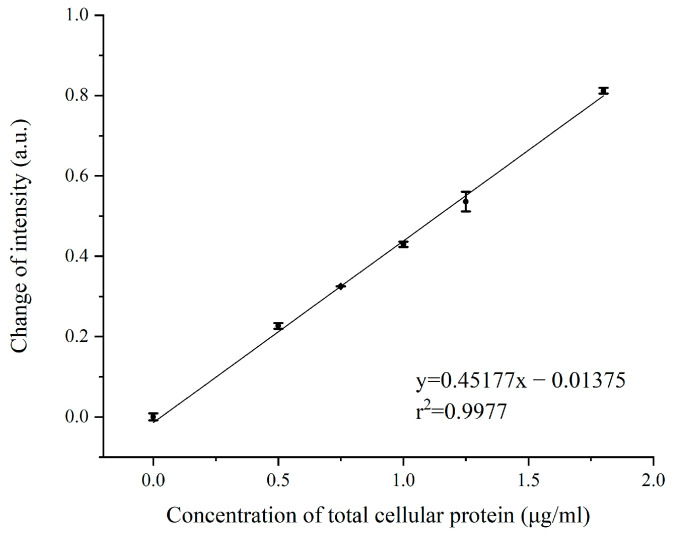
Calibration plot corresponding to the shift of SPR signal with increasing spike protein concentration.

To further examine the relationship between SPR signal intensity and target protein content, serial dilution assay of the EV-T-Dina-2 treatment cellular proteins was performed. Five concentrations of total cellular proteins were analyzed for SPR signal shift by using the C-CASP-3 inhibitor, including 0.00-, 0.50-, 0.75-, 1.0-, 1.25-and 1.75-μg/mL. As shown in Figure 6, a linear correlation was obtained using the least-squares fitting method, revealing the positive linear correlation between SPR signal intensity and expression level of C-CASP-3 in total cellular proteins. The linear correlation coefficient (r2) was calculated as 0.9977, showing that the signal intensity is proportional to the total cellular protein concentration, and thus with the expression levels of C-CASP-3. Therefore, these data show that our ISPRi biosensor can next be adapted for quantitative detection of C-CASP-3 expression in apoptotic cells. 

After optimizing the incident wavelength and angle, the system uses incoherent near-infrared light as the excitation light source and combines the dual 4f optical path to conveniently obtain the optimal excitation angle, achieving an improved RIR to 5.20 × 10^−6^ RIU. However, there is still much room for improvement in system sensitivity. For example, the usage of narrower bandwidth filters may render a sharper SPR angular spectrum. Also, the sensing noise can be reduced by employing a high-brightness light source for SPR excitation. Alternatively, the CMOS with better suppression of dark noise can be used to further improve the SNR of the system.

## 4. Conclusions 

In summary, we developed a high-sensitivity, real-time, and simple ISPRi system. Importantly, this study demonstrated that parallel biosensing could be conducted to detect a target protein in various cell samples under different treatments, without the need for any specific staining or pretreatment of molecules. This provides an efficient, convenient, and highly sensitive means for studying biological processes, such as apoptosis, at the molecular level.

## Data Availability

No new data were created or analyzed in this study. Data sharing does not apply to this article. We used only publicly available datasets for experimentation.
